# Sensing pain: Embodied knowledge in endometriosis

**DOI:** 10.1177/13634593231214938

**Published:** 2023-12-04

**Authors:** Elina Helosvuori, Venla Oikkonen

**Affiliations:** Tampere University, Finland

**Keywords:** chronic pain, clinical encounters, embodiment, endometriosis, experience

## Abstract

The article explores how sensations of pain are turned into embodied knowledge in endometriosis, a chronic gynaecological illness characterized by persistent, possibly paralysing pain. While previous studies have shown how people with endometriosis struggle to achieve accurate diagnosis and effective treatment, we examine the ways in which some of these difficulties are rooted in the complexities of embodied experiences of endometriosis pain and the challenges of translating the sensed patterns and shifts in pain into a language acknowledged within a clinical setting. Building on a phenomenologically inspired approach to chronic pain and drawing on interviews with people diagnosed with endometriosis in Finland, we examine how our interlocutors use their embodied sensations of pain to adapt to the evolving biomedical and lived surroundings in which their pain is evaluated and managed. The analysis shows how living with chronic pain involves constantly attuning to the multitude of symptoms as well as developing personal strategies of communicating sensations of pain to gain medical recognition and care. We argue that while the lived complexities of the body with endometriosis may fall outside the scope of medical practices of measuring, such complexities nevertheless require medical acknowledgment and careful attention.

## Introduction

Chronic pain is notoriously difficult to treat effectively and safely. With its often poorly understood causative mechanisms, it collides with the neoliberal rationalities of cost-effective healthcare and complicates public debates about pain medication addiction. This article addresses the evasiveness of chronic pain as an object of healthcare intervention by analysing the lived complexities of pain in a gendered chronic illness, endometriosis. In endometriosis, cells similar to the uterine lining grow outside the uterus causing persistent, possibly paralysing pain that manifests in the pelvic area as well as in organs and body parts – chest, legs, digestive system – with no direct connection to reproduction. Previous studies have shown how people with endometriosis struggle to achieve accurate diagnosis and effective treatment (Bullo, 2018; Griffith, 2020; Hudson, 2022; Markovic et al., 2008; Seear, 2009). These studies suggest that endometriosis pain is often dismissed as normal period pain or confused, for example, with gastrointestinal or mental health issues leading to significant delays in diagnosis and treatment.

The article explores how people with endometriosis attune to the multitude of pain affecting different parts of their body and turn pain sensations into a form of embodied knowledge. It examines how people with endometriosis draw on this embodied knowledge in deciding when and how to seek medical care and how to navigate everyday life with chronic illness. Drawing on phenomenological approaches to embodiment and illness experience, we ask how the personal embodied histories of living with pain structure the ways in which people with endometriosis conceptualize, assess and manage their pain, and how they seek to translate their pain to healthcare professionals. We highlight that the lived complexities of the body with endometriosis often fall outside the scope of medical practices of measuring pain. We argue that embodied knowledge of endometriosis provides an important contribution to the medical knowledge of endometriosis as a chronic illness.

Focusing on in-depth interviews with people diagnosed with endometriosis in Finland, the article shows how living with chronic pain involves constant observation of the multitude of symptoms across the body. Our analysis identifies four interlinked processes through which sensations of pain are turned into knowledge: (1) attuning to changes in the location and intensity of pain; (2) learning to distinguish between harmless pain and pain that requires medical attention; (3) preparing to communicate the shifting embodied experiences of pain in a way that is recognized in a clinical context and (4) negotiating the discrepancies between embodied experiences of pain and the biomedical practices of verifying and measuring it.

In what follows, we first outline our phenomenologically inspired approach to embodied experiences of endometriosis pain and then introduce our data and analytical methods. The four findings sections focus on the above-listed four processes involved in producing embodied knowledge of endometriosis. We conclude by reflecting on the interrelations of sensing, assessing and managing chronic pain.

## The sensing body and clinical assessment

Chronic pain has been theorized in terms of how it challenges the Cartesian division between mind and body and confounds the codes of morality surrounding sickness and health. For example, Jackson (2005) notes that chronic pain is liminal in the sense that it blurs the lines between disease, symptom, tissue damage and subjective experience. For Jones (2016), the debilitating effects of endometriosis pain on both mind and body indicates that endometriosis should be understood as a disability rather than a privately managed, female health problem. Hallström (in press), in turn, shows how experiences of endometriosis pain take shape in relation to entangled temporalities involving waiting time, cyclical and chronic time, and sedimented time. Beyond these specific strands of theorizing chronic pain and endometriosis, phenomenological approaches address the role of human perception in living with chronic pain. Our contribution is placed in this line of research.

Our article draws on phenomenological interest in the sensing body as the precondition of human experience (see e.g. Merleau-Ponty, 1998). Phenomenological approaches are typically used to analyse embodiment ‘as phenomena that are lived’ (Zeiler, 2020: 384). In line with phenomenological research on pain (see e.g. Geniusas, 2020; Käll, 2013b), we explore how pain is lived with in everyday life, including clinical encounters. However, our study is not methodologically phenomenological. As outlined by Geniusas (2020: 8), phenomenology is concerned with the essential structures without which experience ‘as such’ could not qualify as painful.

Instead of the structures of ‘pure’ pain experience, our starting point is to approach the sensing body as a platform of embodied knowledge. While sensations of pain provide a unique viewpoint into the complexities of living with endometriosis, they cannot substitute other forms of knowledge; different forms of medical knowledge continue to play a pivotal role in endometriosis. For example, recent medical discussions have indicated that endometriosis might, in fact, be more appropriately approached as something other than a gynaecological disease. As one recent article sums it, endometriosis is a ‘chronic, inflammatory, hormonal, immune, systemic and heterogeneous disease’ (Chapron et al., 2019: 667). Pain during menstruation has been linked to the inflammation processes at sites of endometriosis growth across multiple organs (Chapron et al., 2019; Taylor et al., 2021). Pain is also caused by the adhesion of tissues, for example, when endometriosis growth attaches ovaries and intestines together. Furthermore, while there has been a move from surgery to hormonal medication as the first-line treatment, the hormonal-immunological mechanisms underlying different types of endometriosis as well as different hormonal therapies are complex and an object of ongoing research (Vannuccini et al., 2022).

However, as we learned from our data, sensations of pain and other symptoms do not always correlate with the medical severity of the disease, nor is making sense of one’s symptoms a straightforward process. At the same time, prior medical information a person has about their condition shapes how they interpret shifts in embodied sensations of pain. We argue that this complex interplay of discrepancies and alignments across embodied and biomedical forms of knowledge makes it crucial to understand the relations between sensing, assessing and managing pain.

We contribute to the discussions outlined above by analysing sensing and translating pain as knowledge production about endometriosis. The question of shareability of pain has been widely discussed in social research on pain (see Jackson, 2005; Wasson, 2018; Whelan, 2003) and especially in phenomenologically oriented literature (Bustan, 2016; Käll, 2013a, 2013b). Elaine Scarry’s landmark work *The Body in Pain: The Making and Unmaking of the World* is a shared reference point in many of these studies. While Scarry’s (1985) influential work emphasizes how words escape the pain experience, phenomenological reading of her work has also highlighted the possible overlap between bodily damage and linguistic expression, which makes it possible to share certain aspects of pain (see Bustan, 2016). The question of shareability of pain is also significant in relation to our view of pain sensations as embodied knowledge that need to be translated into a language understood by healthcare professionals. Translation is particularly crucial in the diagnosis and treatment of endometriosis, which is increasingly focused on symptoms instead of diagnostic surgery (ESHRE, 2022: 6; Terveyskylä, 2019). However, our interview data indicates that clinicians do not always have adequate tools for symptom-based assessment of endometriosis pain and its implications for personalized endometriosis care.

Compared with phenomenological philosophy of pain, our research has a practical orientation, as we focus on processes of sensing and translating pain. While we foreground the experiences of people with endometriosis, we want to highlight the responsibility of medical professionals and institutions to engage with these experiences and participate in developing means of communicating experiences of pain. This engagement would benefit professionals in providing them tools for diagnosing endometriosis and offering patients the care they need. Our interlocutors note that patients are left with a big responsibility in seeking adequate treatment – to the extent that some of them were apologetic in the interviews if it came up that they had not put a lot of time and effort into searching for medical information. We propose that conceptualizing sensing pain as knowledge production enables a nuanced analysis of how attuning to and interpreting sensations shape the interrelations between the patient and their environment, and how tensions between embodied and biomedical forms of knowledge production are experienced and navigated.

## Data and methods

The article draws on 26 interviews with people with endometriosis collected as part of a larger ongoing project on the treatment of gendered chronic illness in Finland. The interviews were conducted in 2021–2023. The interviews were semi-structured and covered themes such as first symptoms, diagnosis, different treatments, peer support, progression of illness over years and different types of pain. We received written informed consent from all our interlocutors. We followed the ethical guidelines of the Finnish National Board on Research Integrity throughout research design, data gathering, analysis and reporting of the results. The interviews were transcribed verbatim and pseudonymized carefully. Mentions of specific locations and other details not necessary for the analysis were removed to ensure that the examples from the interviews cannot be linked to the interview extracts we use in other research publications.

We found the interlocutors through a call posted on our project website and circulated through social media platforms, including a national patient organization’s networks. As a result, the interlocutors represent a range of ages, from the early 20s to the late 40s, as well as a range of locations, including both larger cities and small towns across the country. Our interlocutors have sought medical help across the public and private healthcare services. The interviews thus reflect the cultures and practices of treating endometriosis in Finland broadly instead of focusing on a specific healthcare site. While this scope allows us to address questions about geographical differences in the treatment of endometriosis across Finland central to the larger research project this article is a part of, it has limitations for the analysis in this article. As we sought participants primarily through widely used digital platforms, the interlocutors who responded to our call are familiar with at least some form of digital technology or some social media sites. Furthermore, our interlocutors all understand Finnish, and thus their position as patients within the healthcare system differs significantly from that of non-Finnish speaking immigrants. A follow-up study focusing on non-Finnish speakers with endometriosis in Finland could provide important knowledge about how endometriosis pain and its medical assessment are experienced by people from different cultural backgrounds.

In analysing the interviews, we located all mentions of pain in the transcripts. We noted how the quality, intensity, location and duration of pain was described, and what role pain played in the embodied experience of illness. We also paid attention to how the interlocutors reflected on communicating their pain to healthcare professionals. As the focus of this article is on embodied experiences of endometriosis pain, we have left out discussions of pain medication and treatment options, which warrant a separate detailed analysis beyond the scope of this article.

Each interlocutor was interviewed by one of the two authors. The analysis of the interview transcripts was conducted jointly. Both authors read the materials and discussed the inclusion of examples, identification of emerging themes and interpretation of quotations. In the case of differences in interpretation, we discussed the premises on which our readings of the material relied on to arrive at a shared interpretation. The analysis began with a tentative identification of broad themes, such as ‘past experiences of pain as a source of knowledge’, or ‘difficulty of fitting experiences of pain within medical frameworks’. With further rounds of reading, we conceptualized these themes as the above-described four processes of attuning to changes in pain, learning to distinguish between harmless and dangerous pain, preparing to communicate pain and negotiating the discrepancies between experiences and biomedical verification and measuring. We now move on to our findings, categorized according to these four processes.

## Attuning to changes in pain

Although endometriosis is a common disease, its etiology and patterns of progression are not well understood within the medical community (Hudson, 2022; Saunders and Horne, 2021). Social scientists studying endometriosis have demonstrated how historical notions of menstruation as inherently painful affect present-day practices of diagnosing – and misdiagnosing – endometriosis (e.g. Bullo, 2018; Griffith, 2020; Hudson, 2022; Seear, 2014). As noted above, however, recent medical discussions also stress non-gynaecological aspects of the disease, such as the inflammation processes in the body and their role in pain experienced, for instance, during defecation or vaginal intercourse. The adhesion of tissues is understood to be another cause of pain, often intensifying during jolting movement, such as when exercising or riding in a car.

In the interviews with our interlocutors, the multitude of endometriosis pain was evident. The central role of attuning to the sensations of the body to understand the complexities of one’s pain emerged as a key part of living with endometriosis. For example, sensing the nuances of pain was described vividly by Liisa, who was in her mid-30s. Having experienced a gradual development of endometriosis symptoms over 20 years, Liisa felt that the illness should have been diagnosed already 15 years ago. Around the time of diagnosis, Liisa’s symptoms included increasingly intense pain around the abdomen, pain during intercourse as well as bleeding unrelated to menstruation. The sensations of pain and the attunement to new symptoms made Liisa seek an ultrasound scan, which revealed an ovary wrapped around the uterus – a not unusual sign of endometriosis. In our interview, Liisa described how the years of living with endometriosis had resulted in an increased familiarity with the variety of pain:

It’s probably quite typical for endometriosis patients that the pain varies a lot. There’s knitting pin pain, which is the feeling that someone stabs you with a knitting pin or a knife, a severe, sudden, piercing pain that hits a specific part of the body. Then there’s radiating pain, which affects a large area and may emanate to the thighs, pelvic area, back, groin, and you can’t pinpoint where it is because it’s everywhere and it’s burning, hot, intense, radiating.

This quotation shows how sometimes the location of endometriosis pain is experienced as clearly defined – localized or piercing pain – and other times it is sensed as radiating across the body – hot, burning pain – with no clear site of origin.

In many interviews, sensing pain was described by our interlocutors as having an analytical dimension. That is, embodied experiences of pain were understood as a form of embodied knowledge production about one’s illness. The following description, also by Liisa, illustrates the significance of attuning to the differences between one’s experiences of pain:

There’s a distinct pain that I have during ovulation. I can point with my finger that the pain is here, in one of the ovaries, and that it’s pulsating and increasingly severe. Then there’s pain that is like a whisper, an inkling of pain somewhere deep around the uterus. The pain might be so slight for days that at times it feels that I might not have any pain. I don’t usually even medicate it because it’s so mild. Then sometimes it may become gradually so intense that it needs to be medicated. Then there is defecation pain, which is not regular but can periodically be so severe that it feels that I might lose consciousness or that I can’t tolerate the pain. And also when I urinate I sometimes have, at the end, a tightening, pressing pain somewhere down around the vagina and uterus. There’s probably something there.

This example shows how sensing is not a passive state happening to a person but involves actively distinguishing between the nuances of pain. Embodied experiences of pain engender knowledge – such as the tightening and pressing pain around the vagina and uterus suggesting that ‘there’s probably something there’ – as well as serve as a basis of action – such as medicating or not medicating pain.

Sensing pain happens in relation to a person’s previous experiences of different types of pain. Many of our interlocutors had ended up in the emergency ward during the worst pain attacks, an experience that many wanted to avoid in the future as it involved hours spent in hospital corridors waiting for relief. These experiences set a framework within which they evaluated new episodes of pain. We heard recurring descriptions of episodes when our interlocutors had to lie on the floor in extreme pain holding a cell phone. Emergency calls were made only in cases where pain attacks did not pass and became impossible to cope with. Those with no prior experience of severe pain attacks often sought help at the emergency ward, whereas experience gained through living through previous attacks enabled the strategy of waiting. Decisions in these emergency situations were made in relation to previous embodied knowledge concerning location, intensity and duration of pain. In this sense, interpreting pain sensations can be understood as a skill that develops over time.

In addition to personal histories of pain, people with endometriosis interpret their bodies in relation to the bodies of other people with endometriosis and their accounts of pain. Our interlocutors gained information about the range of symptoms by following, for example, discussions in online peer support groups organized by patient advocacy actors such as the patient organization. Through such forums, as well as through materials produced by the patient organization, people with endometriosis find relevant information about the embodied effects of endometriosis. For many of our interlocutors, this information had been crucial in interpreting their own situation, leading them to seek diagnosis or better treatment.

Tia, in her late-20s, was one of the interlocutors who conceptualized pain as situated within the broader spectrum of pain sensations. Tia differentiated between ‘proper pain attacks’ (*kipukohtaus*) and what she called ‘pain situations’ (*kiputilanne*):

My worst pains [. . .] have lasted an hour and a half. If we talk about severe period pain, it’s a couple of days. The pain medication takes off some of the pain, but if the period pain is really bad, the [prescribed] 800 milligrams of [a common brand of ibuprofen] and one gram of [a common brand of paracetamol] remove perhaps ten percent of the pain. Then there are times when I lie on the couch under a blanket with two hot water bottles like a hotdog inside a bun. And then there are smaller pain attacks, I don’t know if you can even call them pain attacks because they are so ordinary [. . .] Someone with more severe pain might be offended if I called them pain attacks – they are more like pain situations.

In this excerpt, as in several other interviews, pain is made sense of in relation to the perceived severity of other people’s endometriosis pain, as suggested by the distinction between pain attack and pain situation. Yet, the sensing body emerges as the primary environment within which a person with endometriosis needs to operate in. The gradations of endometriosis pain are conceptualized through embodied evaluations of, for example, the reduction in pain following a specific medication in the quotation above. At the same time, attuning oneself to the multitude of pain sensations enables coping strategies to emerge. These strategies include, for example, lying on the couch after lunch to release gas from the intestines to avoid pain in the digestive system.

Attuning to sensations of pain also enables embodied evaluation of the potential progression of endometriosis. This connection between sensing a change in the quality, intensity or location of pain and a suspected development in the illness itself came up in several interviews. For example, Laura, now in her mid-40s, provided a description of past personal experiences of pain:

When I think about those times, the first thing [I remember] is the tearing pain in, for example, the rectum, when you notice that there is [endometriosis] growth again, that the tearing pain reaches the entire leg. Then I just wondered what would be discovered if I had an ultrasound or MRI scan now. Then there have been some occasions of pain around the ovaries which made me see a doctor, it felt like being pinched, but it disappeared on its own.

In Laura’s account, attuning to changes in sensations of pain engenders knowledge about the possible appearance of new endometriosis lesions and their location. However, the connection between the changes in pain and the extent of the growth of endometriosis lesions is not straightforward. In this case, the pain around the ovaries experienced by Laura did not reveal changes in endometriosis in medical examination. Yet, in Laura’s account as well as in many of our other interviews, continuous self-assessment of pain appears as a crucial source of knowledge for making decisions about when to seek clinical advice.

## Learning to distinguish between harmless and dangerous pain

In our interview data, learning to know one’s pain appears as a crucial step in distinguishing between pain that may be debilitating but not dangerous and pain that demands immediate medical attention. Attuning to the nuances of pain thus emerges as necessary for managing pain safely. The importance of knowing when pain is safe was highlighted by Maria, who was in her late-20s and lived in a sparsely populated area over 150 kilometres from the closest hospital and 30 kilometres from a local health centre. Wanting to avoid such long and painful trips, Maria considered pain as an unavoidable life companion and emphasized the importance of learning to manage pain adequately and safely. Maria suggested that the most important thing when living with pain was that ‘you begin to recognize the things that trigger pain attacks’ and then ‘A. choose to avoid them, or B. medicate yourself in advance’. Maria had developed the following strategy:

My hobby is riding a snowmobile. I have a freeride snowmobile. Jolting movement is not good for endometriosis, in other words, I myself make the decision that if I ride on the fell I will be ill in the evening. I could choose not to ride, but I’ve decided that I will. So, in practice, I take pain medicine beforehand and in the evening I wear some nice loosely fitting clothes and look like I’m pregnant. I treat the pain with heat and cold and so on.

Before deciding on this strategy, however, Maria had checked with a doctor that ‘the pain you experience doesn’t make the disease worse’. This highlights that the medical infromation a person with endometriosis has received structures the ways in which they interpret their sensations of pain. While having to medicate pain pre-emptively was not ideal, knowing that the pain caused by jolting movement was not dangerous made Maria’s strategy feasible. Ignoring or numbing one’s pain, then, may constitute a way of living with endometriosis when balanced with long-term observations of one’s body and an understanding of the relationship between different types of pain and the progression of illness.

At the same time, paying attention to possible changes in the quality of pain remained important for our interlocutors. For example, some types of pain were taken as signs of possibly dangerous progression of endometriosis. Many of our interlocutors had experienced a variety of difficulties related to digestion and defecation. While these difficulties were often connected to the normal process of intestinal movement, changes in gastrointestinal pain were also interpreted as an indication of the disease getting more serious, that is, as a ‘red flag’ that requires seeking medical evaluation.

This was the case with Julia, who had undergone a type of surgery known as ‘the radical’ in which the uterus and ovaries are removed to stop the progression of endometriosis. In the surgery conducted 2 years before our interview, a part of Julia’s colon had been removed because Julia’s ‘insides were a mess’. The surgery had improved Julia’s pain, although there was no guarantee that it would end endometriosis symptoms. Some time after the surgery, Julia had again begun to feel pain and had an MRI scan. As nothing alarming was found, it was concluded that the pain was likely related to intestinal movement. At times, defecation indeed caused ‘paralyzing pain’. With the help of a pelvic floor physiotherapist, Julia had taken action to alleviate the situation. However, in their latest session, the physiotherapist had wondered whether there was after all something wrong with the ‘intestine seam’ constructed in the surgery. If the seam had thinned, there might not be enough room for foeces to move through the intestines. The physiotherapist’s hunch corresponded with Julia’s own sensory experience:

I have occasionally experienced sensations that you could call pain in the same place where the intestine seam is. A few times there has been pain that is incapacitating right here, the seam is here in the lower left side. That has made me wonder what there is.

To improve the functioning of the digestive system, Julia took fiber supplements and tried to defecate without pushing as instructed by the physiotherapist. If the situation nevertheless worsened, the next step would be another gastrointestinal operation – something Julia wanted to avoid. Julia had the impression that surgical interventions to the seam would constitute a risk of it ‘popping’. ‘I don’t want that’, Julia concluded. Instead, Julia aimed to attune to embodied sensations and to interpret what they suggested about the condition of the seam. To do this, Julia drew on both the capacities of the body to sense pain and prior medical information about the presence and location of the seam.

These examples show that although pain might not be dangerous per se, being able to assess whether sensations of it should be taken as alarming signals requires the ability to sense pain. This ability might even be framed as an asset in the management of endometriosis, as in our interview with Laura:

I’ve actually used very little pain medicine to treat this endometriosis because I’ve wanted to know how I feel. I find it scary to use [a common brand of ibuprofen] endlessly because of side effects but also because I think it’s scary to remove the pain as then I might not notice something.

This quotation demonstrates that sensitizing oneself to pain in its myriad forms and locations constitutes an important means through which the overall progression of the illness is traced. Here, as in several other interviews, the body in pain is not easily or fully knowable and thus all sensations need to be carefully observed and accounted for.

## Preparing to communicate pain

When people with endometriosis experience symptoms that they consider alarming and seek medical advice, they face a challenge: how to translate the sensations of pain to the clinician? Likewise, those seeking endometriosis diagnosis need to articulate the specific nature of their pain that distinguishes it from period pain. Previous research has shown that people with chronic pain often struggle to find ways of conveying successfully their embodied observations of pain, and that clinical encounters may leave patients feeling that the extent of their pain symptoms has been misunderstood (Denny, 2009; Hudson, 2022). Some studies have documented how patients deal with such challenges. For example, Bullo (2020) analyses the central role of metaphor in how people with endometriosis conceptualize and communicate their pain. Bullo identifies metaphors likening endometriosis pain to physical injury (such as ‘stabbing pain’), metaphors portraying pain in terms of volume, pressure, temperature or weight and metaphors depicting pain as a force that transforms a person. Furthermore, even when pain is acknowledged by medical professionals, people with chronic pain may resist a clinician’s interpretation of its causative mechanisms. Drawing on observations at a pain clinic, Declercq (2023) identifies moments of misalignment between patients’ and clinicians’ views of the extent to which different physiological, psychological and social factors contribute to the patients’ chronic pain.

The difficulty of communicating pain is also a central theme in endometriosis patient advocacy and peer support. For example, the Finnish patient organization’s website provides advice for people diagnosed with endometriosis or those seeking diagnosis as to how to prepare for doctor’s appointments. According to the website, especially during severe pain attacks or visits to an emergency ward, it might be difficult to verbalize the specific qualities of the pain, which is crucial information to guarantee adequate care. In such emergencies, a written-down list of medications, symptoms and previous knowledge about the location and extent of endometriosis lesions may be helpful.

The difficulty of translating the nuances of pain to medical professionals is visible in our interviews. Many of our interlocutors felt that they must fight to get the help they need to cope with pain. To convince the clinicians of what the patients knew about their own pain, some of our interlocutors told us that they wrote in-depth descriptions and diaries of their symptoms before doctor’s appointments or surgical procedures. Despite these efforts, many ended up feeling disappointed and neglected, as they felt that the clinician barely glanced at the documentation of symptoms they had painstakingly produced.

Among many of our interlocutors, the embodied histories of living with endometriosis lingered to the encounters in which the inner experience is translated to the external medical environment. Our interview with Eeva shows how the translation process is important not only in terms of adequate medication but also in terms of diagnosis. Having suffered from gastrointestinal pain for several decades, Eeva’s endometriosis was diagnosed around the age of 40 in connection to fertility treatments. One of Eeva’s early symptoms had been gastrointestinal pain and diarrhea around the time of menstruation. However, at the time of our interview, Eeva’s gastrointestinal pain had become nearly ever-present:

The endometriosis has got out of control and is no longer connected to periods. The symptoms can certainly be worse then [during menstruation]. I think I can recognize that. Now I’m wondering what is endo pain and what is gastrointestinal pain because they are in a way the same thing. I just almost said that I don’t have endo pain but gastrointestinal pain but they are the same in this case [laughs].

According to Eeva, a diagnosis might have been possible years ago if someone had linked the gastrointestinal pain to endometriosis. At the same time, even after years of living with gastrointestinal pain, Eeva still found it challenging to conceptualize the gastrointestinal pain as a manifestation of endometriosis. Crucially, there are several other interviews in our data in which either clinicians or patients – often both – have dismissed gastrointestinal pain as not related to endometriosis.

The need to translate embodied experiences of pain into a language understood by healthcare professionals had led many of our interlocutors to develop sophisticated strategies to prepare themselves for medical encounters. Anna, in the late 40s, told us:

Sometimes when I have more energy I plan a careful strategy that includes writing down everything I need to say, what I should not say, what at least I have to get through. It’s almost like talking to a journalist [about your own work], things you need to get through in an interview. It doesn’t matter what the journalist says, you just repeat your points. I have a similar strategy with doctors.

Planning communication strategies to communicate pain was a common theme across several interviews. Yet, such strategies may be difficult to follow during the worst symptoms. Anna told us: ‘When I’m exhausted and really need help, the strategy may not work because I’m too tired and psychologically burned out that the negotiation just exhausts me so much that I can’t’.

Furthermore, the lingering, bitter disappointments experienced in past clinical encounters affect the ways in which our interlocutors seek to communicate their pain in medical encounters. Past disappointments in seeking help linger and shape how patients experience these situations. For example, Sofia, in the early 30s, told us that she only ‘gradually believed it’ when, after years of symptoms, a new doctor finally verified that ‘yes this is endometriosis and it’s visible in the ultrasound and I can also feel the lesions with my hands’.

## Negotiating the discrepancies between experiences and biomedical verification and measuring systems

When endometriosis is diagnosed or its progression is assessed in the clinic, there are established medical tools of measuring that clinicians may use to interpret the symptoms described by the patient. Two sets of practices of assessing pain are particularly central in clinical settings: the use of pain scales to evaluate the severity of the pain and the use of imaging technologies to establish a connection between experiences of pain and a visualizable pathology inside the body. Through these practices, clinicians try to establish the medical significance of the patient’s pain – for example, whether a certain type of pain is linked to endometriosis or another condition, and whether a new type of pain indicates a worsening of endometriosis. However, our interview data, along with previous research on experiences of endometriosis, suggest that medical tools of measuring pain cannot easily capture the nuances and complexities of persistent pain.

Writing in the context of phantom limb pain, Middleton (2022) has developed the concept of ‘datafication of pain’ to describe the process through which pain as subjective, embodied experience is translated into a presumably quantifiable entity and, subsequently, enacted as an object of biomedical intervention. Middleton notes that pain assessment questionnaires seek to circumvent the problem of the subjectivity of pain sensations by focusing on changes in a person’s pain over time. However, Middleton notes that such pain scales inevitably exclude relational and contextual aspects of pain – for example, how pain sensations fluctuate in relation to social situations and personal embodied histories of living with pain. In the context of endometriosis, Denny (2009) has noted that pain scales used by clinicians focus on particular aspects of pain. Denny’s interlocutors in the UK ‘were asked to score different types of pain associated with endometriosis, e.g., pelvic pain or dyspareunia, but were rarely asked about the quality or duration of the pain, or the effect of the disease on their ability to function or on their relationships’ (Denny, 2009: 990). Likewise, Whelan (2003) has analysed differences between a pain scale developed by clinicians and a pain scale developed by an endometriosis activist. The activism-based scale addresses the problem of communicating the subjectivity of endometriosis pain by assessing, for example, the extent to which the pain limits a person’s ability to function in everyday life while also listing pain medications and their effects on the pain (Whelan, 2003). These studies clearly show that how pain is measured affects how embodied sensations of pain can operate as a basis of knowledge production about the presence or progression of endometriosis.

In our data, the clinical use of scales to standardize and assess sensations of pain is mentioned. At the same time, the difficulty of translating pain into charteable and quantifiable data is evident. For those living with pain daily, there may be considerable differences between days: days off from work are often easier than working days during which pain may be constantly undulating through the body. Furthermore, through years of living with gradually evolving pain, many have accepted a certain level of moderate pain as a life companion whose presence is noted but not reacted to.

Our interview with Eeva demonstrates how the embodied histories of living with endometriosis pain shape how someone with endometriosis may respond to a request to score their pain. Describing postoperative pain after endometriosis surgery, Eeva notes:

Pain is a difficult issue because it’s so subjective. You can use a VAS [visual analogue scale] or such – that’s what they did in the hospital when I woke up after the operation, what is the pain like on a VAS with a scale from one to ten. I kept saying three, but I couldn’t move because I felt so sick. Being used to pain skews your own assessment. When I realised I should say four I started getting medication.

The pain VAS discussed in the excerpt is one of the established clinical forms of documenting pain in which the patient is asked to rate their pain intensity on a continuum between none and extreme pain, sometimes – like here – with the help of numbers. Eeva’s experiences at the hospital highlight that the ways in which pain scales are put to use may reinforce rather than remove barriers between an embodied pain sensation and clinical verification: ‘If someone cuts across your stomach with a knife then you just walk in a stooped position, but on another person’s VAS that would be an eight.’ Eeva’s description also shows how the task of translating personal embodied experiences of pain may involve purposely sidestepping one’s own sense of the spectrum of possible pain and instead figuring out, sometimes through trial and error, how pain scores are interpreted by healthcare professionals.

In addition to pain scales, imaging technologies such as ultrasound and MRI are used to verify the existence of pain by trying to link it to a pathology. The current treatment guidelines for endometriosis in Finland consider excessive period related pain as a sufficient basis for tentative diagnosis, a situation that reflects the overall transition from surgery to hormonal treatment in endometriosis (ESHRE, 2022: 6; Tiitinen, 2022). However, imaging tests continue to play a central role in diagnosis. In particular, ultrasound examinations are commonly conducted during a gynaecologist’s appointment. At the same time, it is known that certain forms of endometriosis may be hard to detect with available imaging technologies (ESHRE, 2022: 6; Terveyskylä, 2019). This role of imaging technologies as a means of verifying endometriosis is present in our interview data. While many of our interlocutors hoped to have their symptoms verified through a scan, they were ambivalent about the relationship between knowledge produced through imaging technologies and knowledge produced through embodied sensations of pain. In particular, past experiences of ultrasound scans that did not establish a connection between pain and endometriosis lesions shape expectations about future clinical encounters, including both hopes and worries about whether an evolving pain will be verified.

Pain symptoms that reappear after radical surgery constitute a particularly charged area. While pain may be a result of nerve damage or surgery-related scarring, endometriosis tissue that remains in nonreproductive organs can, in some circumstances, cause endometriosis symptoms. Our interlocutor Roosa, in the late 40s at the time of the interview, describes visiting a gynaecologist after radical surgery:

I went to the gynecologist’s to talk about the pain medications and other things. The gynecologist examined me and said that there’s no endometriosis anymore, nothing visible with the ultrasound, and because there’s no endometriosis there can’t be pain, and if there’s pain, it will pass so quickly that there’s no reason to take medication. My own view was quite different, okay perhaps there are no endometriosis lesions but something is causing the pain. It can’t be that I’m just imagining the pain.

In Roosa’s case, the use of imaging technology does not resolve the gap between embodied sensations of pain and medical knowledge. Instead, it engenders a sense that the lived complexities of the body with endometriosis may simply be outside the scope of medical knowledge production. While it is always difficult for patients to challenge imaging tests that do not reveal clear pathologies, it can be particuarly difficult in the context of the medical understanding of radical surgery as an effective solution to severe endometriosis. That those undergoing radical surgery have often developed a strong sense of what their endometriosis feels like reinforces the perceived gap between embodied experience and visual clinical evidence as modes of knowledge production.

The absence of a visualizable or measurable pathology does not necessarily mean that endometriosis symptoms would go untreated. Many of our interlocutors who were diagnosed with endometriosis on the basis of their symptoms (rather than an ultrasound or MRI scan) had medication to help with pain and a hormonal product to slow down the progression of the illness. However, the absence of visual verification left many with a sense of uncertainty about their status or identity as someone with endometriosis.

## Conclusion

The interviews analysed in this article highlight the importance of understanding sensing of endometriosis pain as embodied knowledge production. The four processes of attuning to, distinguishing, translating and measuring pain we have identified testify to the complex interrelations of sensing, assessing and managing pain in the treatment of endometriosis. They also show that it is crucial to envision ways in which sensory existence – or aspects of it – could be merged with clinical standards of treatment. Approaching sensing pain as a form of knowledge production provides a means of understanding the question of the shareability of pain as situated, pointing to not only disconnections but also patterns of alignment across embodied and medical knowledges. As suggested earlier in the article, there is need for further research to understand the role of different cultural backgrounds in how endometriosis pain and its medical assessment are experienced by those seeking care within the Finnish healthcare system.

Our analysis suggests that it is important that healthcare professionals and people with endometriosis navigate between different forms of knowledge production to bring together subjective experience and clinical evidence. While finding a plausible explanation for pain verifies the medical actions taken to tame the pain, it is also essential for the sensing patient who may find it exhausting to constantly fight for adequate treatment. Yet, medical tools of measuring pain cannot easily capture the nuances and complexities of persistent pain.

Our analysis shows that sensing is not a passive state but involves actively distinguishing between the nuances of pain: its intensity, quality, location and pattern of development. Sensing has an intuitive dimension, but is also learned through trial and error, by contrasting one’s past pain experiences and past personal knowledge of how pain sensations correspond with the progression of illness. Sensing is also shaped by the person’s prior medical knowledge of their conditon. Furthermore, assessing personal sensations of pain takes place in relation to what is known – or assumed – about the intensity of other people’s endometriosis pain.

We have shown that embodied sensations of pain serve as a basis for action. Recognizing changes in pain intensity, type or location that could indicate progression or a new turn in the illness is a central aspect of living with endometriosis. Our interlocutors describe processes of constant assessment over whether pain is safe (and can thus be managed with pain medication and ignored as much as possible) or whether it requires medical attention. However, significant uncertainties remain, as the connection between pain sensations and a pathology – such as new endometriosis lesions – is often unclear even after years of living with endometriosis.

The analysis also demonstrates that the process of translating pain in medical encounters is affected by personal lingering sensory histories. According to our findings, patients enter a doctor’s office bearing the sediments of pain with them. They might also be haunted by past experiences of failure in gaining medical help such as scans or examinations that did not verify a connection between debilitating pain and a physical pathology. Our analysis thus highlights the importance of understanding the embodied histories of pain as a part of not only the everyday management of illness but also clinical encounters where pain is translated, measured and verified. We propose that the tension between the growing emphasis on symptoms as the basis of treatment and the long-standing epistemic privileging of visual verification as ‘real’ evidence needs to be addressed in the clinical settings where endometriosis care is sought and offered. While the lived complexities of the body with endometriosis may fall outside the scope of medical practices of measuring, such complexities nevertheless require medical acknowledgment and careful attention.
